# Implementation of Adolescent-Friendly Voluntary Medical Male Circumcision Using a School Based Recruitment Program in Rural KwaZulu-Natal, South Africa

**DOI:** 10.1371/journal.pone.0096468

**Published:** 2014-05-02

**Authors:** Carl Montague, Nelisiwe Ngcobo, Gethwana Mahlase, Janet Frohlich, Cheryl Pillay, Nonhlanhla Yende-Zuma, Hilton Humphries, Rachael Dellar, Kogieleum Naidoo, Quarraisha Abdool Karim

**Affiliations:** 1 Centre for the AIDS Programme of Research in South Africa (CAPRISA), Nelson R Mandela School of Medicine, University of KwaZulu-Natal, Durban, South Africa; 2 ZimnadiZonke, Pietermaritzburg, South Africa; 3 Mailman School of Public Health, Department of Epidemiology, Columbia University, New York, New York, United States of America; University of Cape Town, South Africa

## Abstract

**Background:**

Epidemiological data from South Africa demonstrate that risk of human immunodeficiency virus (HIV) infection in males increases dramatically after adolescence. Targeting adolescent HIV-negative males may be an efficient and cost-effective means of maximising the established HIV prevention benefits of voluntary medical male circumcision (VMMC) in high HIV prevalence–, low circumcision practice–settings. This study assessed the feasibility of recruiting male high school students for VMMC in such a setting in rural KwaZulu-Natal.

**Methods and Findings:**

Following community and key stakeholder consultations on the acceptability of VMMC recruitment through schools, information and awareness raising sessions were held in 42 high schools in Vulindlela. A three-phase VMMC demand-creation strategy was implemented in partnership with a local non-governmental organization, *ZimnadiZonke*, that involved: (i) community consultation and engagement; (ii) in-school VMMC awareness sessions and centralized HIV counselling and testing (HCT) service access; and (iii) peer recruitment and decentralized HCT service access. Transport was provided for volunteers to the Centre for the AIDS Programme of Research in South Africa (CAPRISA) clinic where the forceps-guided VMMC procedure was performed on consenting HIV-negative males. HIV infected volunteers were referred to further care either at the CAPRISA clinic or at public sector clinics. Between March 2011 and February 2013, a total of 5165 circumcisions were performed, the majority (71%) in males aged between 15 and 19 years. Demand-creation strategies were associated with an over five-fold increase in VMMC uptake from an average of 58 procedures/month in initial community engagement phases, to an average of 308 procedures/month on initiation of the peer recruitment–decentralized service phase. Post-operative adverse events were rare (1.2%), mostly minor and self-resolving.

**Conclusions:**

Optimizing a high volume, adolescent-targeted VMMC program was feasible, acceptable and safe in this setting. Adaptive demand-creation strategies are required to sustain high uptake.

## Introduction

KwaZulu-Natal in South Africa is at the epicentre of the global human immunodeficiency virus (HIV) epidemic with an estimated 24.7% infection prevalence in the general population in 2011 [1]. The rural Umgungundlovu district in KwaZulu-Natal is one of the three highest HIV disease burden districts in South Africa and is a priority district for HIV prevention intervention efforts [2], [3]. Surveillance data in the district and elsewhere consistently demonstrate that in males HIV infection risk increases dramatically following adolescence, with prevalence in boys aged 15–19 estimated at 2–3%, compared to 11–12% in those aged 23–24 years [4], [5].

Voluntary medical male circumcision (VMMC) has been demonstrated to reduce risk of HIV acquisition through heterosexual vaginal sex in males by 50–73% [6]–[9], and is an essential part of the ‘HIV prevention toolbox’ of evidence-based behavioural, biomedical and structural interventions [10]–[12]. Targeting VMMC at young males as part of such a combination prevention program in schools before entry into the high risk period could represent a directed and cost-effective means of altering current epidemic trajectories for HIV. An adolescent-targeted strategy could also represent the ‘path of least resistance’ in terms of VMMC scale-up, considering several studies have shown that uptake of VMMC is highest in this age group [13], [14]; indeed, younger men are less likely to be inhibited by barriers to VMMC reported by older men which include concerns about taking time off work and abstaining from sex following the procedure [15].

Circumcision is not a traditional practice in Umgungundlovu and although several studies show general acceptance of the procedure in sub-Saharan Africa [16]–[19], local community responses to an adolescent-targeted VMMC program are unknown, and no school-specific framework for demand creation and implementation has been developed. The purpose of this pilot conducted by the Centre for the AIDS Programme of Research in South Africa (CAPRISA) was to assess the feasibility, acceptability and uptake of VMMC services when combined with novel demand creation strategies in high school students in the Umgungundlovu district.

## Methods

### Ethics statement

VMMC is part of routine service provision in South Africa and implementation is in accordance with World Health Organization (WHO) guidelines [20], [21]. The VMMC services described in this study were provided as part of routine public health program in the district. Consent for the VMMC procedure was obtained in accordance with Section 12(9) of the Children's Act in South Africa which requires that boys aged 16 or older must provide individual consent and that boys aged younger than 16 must obtain parental consent [22]. Ethical review was sought and granted by the Biomedical Research Ethics Committee of the University of KwaZulu-Natal for chart review of clients utilizing the CAPRISA VMMC services that forms the basis of this manuscript (BE286/12).

### Populations

Recruitment was initiated in all high-schools in Vulindlela, a rural sub-district of Umgungundlovu. The school calendar year runs from January to December with the majority of high school students aged between 11 and 20. The school holidays include one week at the beginning of April, three weeks at the beginning of July, one week at the beginning of October and a four to five week summer holiday from December until mid-January. All 42 high schools in Vulindlela were targeted, a total male population of 11,088. The high-schools were all mixed sex, day schools and the number of males per school ranged from 91 to 892 with an average per school of 317. Male students were recruited for VMMC between March 2011 and February 2013. The target was to achieve 70% VMMC coverage in these schools, which represents 7761 procedures. The target age group was 16 to 20 years, but services were also available to younger volunteers aged 12–15 years who had parental consent and to out of school volunteers over 20 years old.

### Optimization of an adolescent-friendly VMMC-demand creation strategy

A three phase VMMC-demand creation strategy was implemented in partnership with a local non-governmental organization (NGO), *ZimnadiZonke* that involved: (i) community consultation and engagement; (ii) in-school VMMC awareness sessions, centralized HIV counselling and testing (HCT) service access and VMMC service access facilitation; and (iii) peer recruitment and decentralized HCT service access. In each of the 42 high-schools a teacher was identified by the school principal to serve as the liaison with the VMMC recruiters. The role of this teacher was to provide oversight of the VMMC program in the school and to alert CAPRISA should there be any problems. The teachers were not actively involved in the recruitment of students for VMMC.

#### Phase one: Community consultation and engagement

To assess community support and acceptance of the proposed program, consultations were held with traditional-, school-, and community opinion- leaders over a seven-month period from June 2010. Following these meetings and with additional consultation, consent and collaboration with the community research support group, an outreach plan for information dissemination was developed and a partnership established with *ZimnadiZonke*. Community information sessions were provided in churches and other small informal community groups. The information session was focussed on explaining the evidence that VMMC prevents HIV acquisition and what the VMMC procedure involved. Further, general community outreach focused on VMMC/HIV information sessions held at primary health care clinics for patients in waiting rooms. Regular feedback was provided to the community through the community research support group and other community events.

#### Phase two: In-school VMMC awareness sessions, centralized HCT service access and VMMC service access facilitation

From May 2011, four VMMC coordinators were appointed to disseminate information on the HIV risk and the preventative benefits of VMMC and local VMMC service points during school assemblies. Students were also provided with information on VMMC as part of the sexual and reproductive health information sessions during Life Orientation classes. HCT services to students were offered in schools by counsellors from *ZimnadiZonke* and referrals made for volunteers to the CAPRISA clinic for the surgical procedure with transport to and from the school provided if needed. Information and HCT services in school took place on Monday toThursday and the surgical procedure was undertaken on a Friday afternoon and Saturday and post-surgical assessments were done by nurses in school to minimize disruption of schooling.

#### Phase three: Peer recruitment and decentralized HCT service access

From March 2012, early student adopters of CAPRISA VMMC services were invited to be peer recruiters, informing fellow students about the benefits of VMMC and explaining the surgical procedure. Peer recruiters were also responsible for scheduling appointments for VMMC and co-ordinating transport to the CAPRISA VMMC clinic. The clinic staff had to be careful to incentivize the peer recruiters in a way that did not lead to coercion of adolescents for VMMC. Detailed training was provided to the recruiters on the VMMC procedure, how to recruit males interested in VMMC, supervision of the volunteers on the day of the clinic, and ensuring that boys requiring parental consent obtained the consent before attending the clinic. Small prizes such as watches, toiletries and t-shirts were provided to recruiters during the training. In order to fulfil their duties the recruiters were provided with airtime vouchers (not more than $3 equivalent per recruiter) for cell-phones; name badges and T-shirts to aid identification of legitimate recruiters. At the end of the program an awards ceremony was held to acknowledge the performance of the recruiters and the teachers at which certificates were presented and medals and trophies presented to the best performing recruiters and schools. In this phase, HCT was undertaken at the CAPRISA clinic as well as other public sector clinics closest to the schools where there was demand. Post-surgical assessments were undertaken in schools the following week.

### Optimization of adolescent-friendly VMMC procedures

A VMMC service was established at the CAPRISA clinic that offered: (i) HCT to all male volunteers; (ii) VMMC to males who tested HIV negative and (iii) referral to care to males who tested HIV positive. In areas where transport to the CAPRISA VMMC site was a challenge, temporary CAPRISA-staffed VMMC clinics were established in public health facilities. The CAPRISA VMMC clinic procedures and schedule were adapted throughout the program in order to best meet the service demand and to provide an “adolescent friendly” service.

#### Schedule optimization

Initially, the CAPRISA VMMC clinic operated on weekdays during school hours; students were offered after-school access to HCT services provided by *ZimnadiZonke*, in mobile tents erected on school premises. From May 2011 recruitment and HCT was provided in-school from Monday to Thursday and referrals were made for transport to the CAPRISA VMMC clinic on Fridays and Saturdays, to facilitate student access and reduce disruption to school timetables. In the final optimization in March 2012, recruitment continued in schools but HCT and VMMC services were offered at the CAPRISA VMMC clinic on a Saturday; transport to the clinic was provided. Post-operative review services were provided by a team of visiting nurses in-school to further minimize disruption to learning.

#### Clinic procedures

All males over 12 years with appropriate consent were eligible for VMMC services. Circumcision was not performed on males who tested positive for HIV on the day of the scheduled VMMC procedure as suitability to undergo surgery could not be determined until CD4 and viral load results were obtained. Therefore, these males underwent a mock process to maintain confidentiality and reduce stigma and discrimination and were referred to CAPRISA's AIDS treatment program. The mock process was considered ethically necessary in order to prevent social harm to the males through unintentional disclosure of their HIV status on the day of the VMMC clinic visit. In addition to HIV, volunteers were screened for other sexually transmitted infections (STIs) and if necessary treated according to syndromic management of STIs as per Department of Health guidelines; VMMC was rescheduled for those males who successfully completed STI treatment.

All males undergoing the VMMC procedure were required to provide written consent if over 16 years or written parental consent and a statement of assent if younger than 16 years. Initially, boys younger than 16 years were given informed consent forms to take home to be signed by their parents. However, as part of the optimization of the operating procedures for the clinic, parents were later provided with teacher-facilitated VMMC information sessions at the schools, at which they were requested to provide consent should they wish their child to be circumcised. Elements of the WHO's Models for Optimizing the Volume and Efficiency (MOVE) of VMMC were employed to maximize efficiency [23]. Circumcisions were performed by qualified clinicians using the forceps guided method [20], under nurse-administered local anaesthetic. Sterilized disposable circumcision kits and electro-cautery were used to reduce the time required per procedure and improve program efficiency.

After the procedure, students were provided with sufficient analgesics for two days of pain relief and were counselled on the requirement for a six-week period of abstinence. Post-operative follow-up was conducted in accordance with the WHO guidelines [20], [21]. During follow-up, students were offered HCT and asked to complete a questionnaire to evaluate the VMMC service. They received an emergency contact number for discussion of any post-operative problems. Teachers were identified in each school to work with peer recruiters in surveillance for any complications which required involvement of clinical staff. Any out of school client who underwent VMMC at the clinic was requested to attend the clinic for follow-up.

## Results

**Figure 1:**
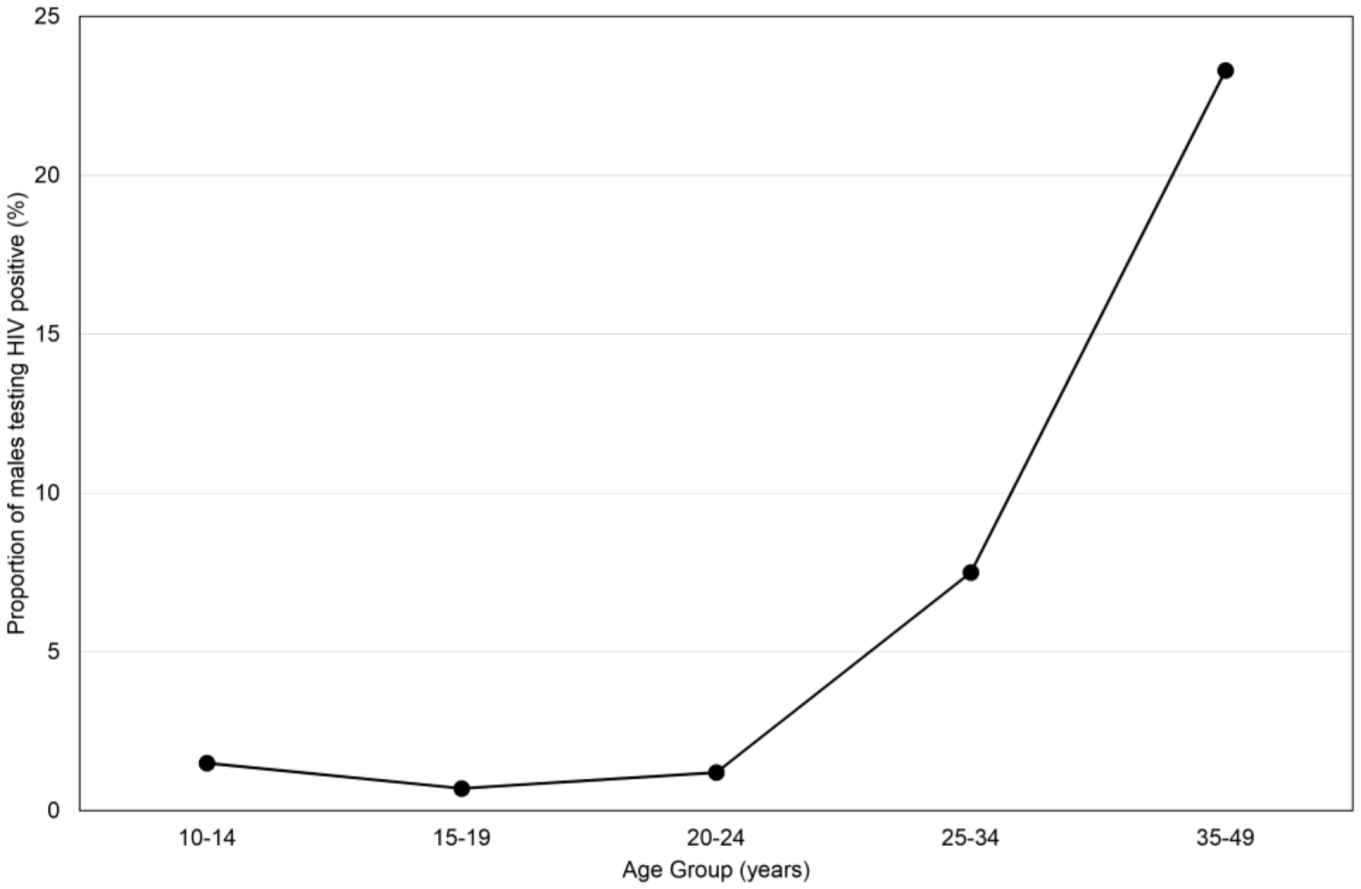
Proportion of males testing HIV positive in pre-VMMC HCT by age group.

The results of the pre-VMMC HCT confirmed very low HIV prevalence in males up to 20 years of age, and a rapid increase thereafter. The proportion of males who tested HIV positive was 1.5% in the 10–14 age group, 0.7% in the 15–19 age group, 1.2% in the 20–24 age group, 7.5% in the 25–34 age group and 23.3% in the 35–49 age group (Figure 1).

**Figure 2:**
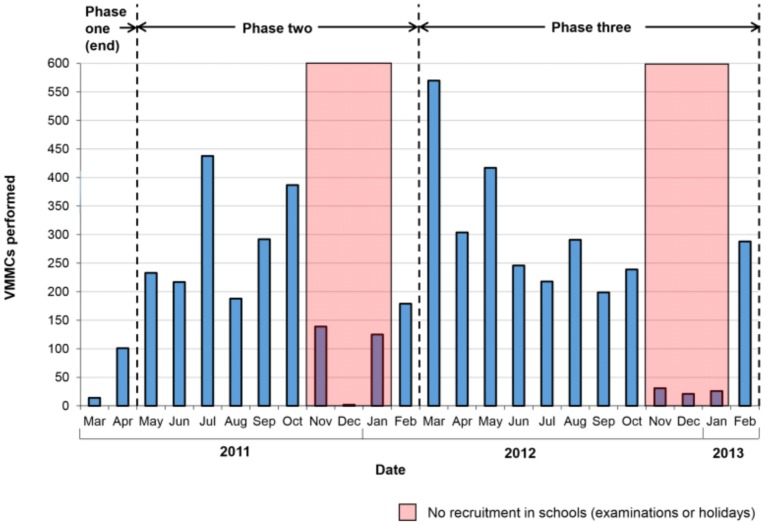
VMMCs performed by the CAPRISA VMMC program between March 2011 and February 2013. VMMCs performed by the CAPRISA clinic per month shown. No recruitment was undertaken in schools in November and December at the request of school officials to avoid disruption to examinations; recruitment was also not undertaken in the school holidays in January. Phase 1 of demand creation (community consultation) began in June 2010 and continued until April 2011. Phase 2 of demand creation (in-school information dissemination) was initiated from May 2011 to the end of February 2012. Phase 3 of demand creation (peer recruitment) began in March 2012 and continued until the February 2013.

Over 24 months from March 2011, the CAPRISA VMMC clinic performed a total of 5165 circumcisions (Figure 2). This total is estimated to crudely represent 47% (5165/11088) coverage in the 42 high schools. In the first two months of the clinic opening, only phase one of the demand creation strategy was implemented, and early adopters contributed an average of 58 procedures/month. Due to the low number of males attending the primary health care clinics, and therefore accessing the VMMC information systems, it was decided to try providing VMMC information sessions in schools and supporting this by appointing VMMC coordinators in each school. This initiative of active recruitment at schools in phase two of the demand creation strategy in May 2011 increased uptake of VMMC more than four-fold, to an average of 276 procedures/month (excluding periods of non-recruitment). Following implementation of phase three of the demand creation strategy in March 2012, which involved active peer recruitment, the average uptake increased further to 308 procedures/month (excluding periods of non-recruitment). Decreases in monthly levels of VMMCs performed can largely be explained by periods of school holidays (January) and requests from schools not to recruit during school examination periods (November and December).

**Figure 3:**
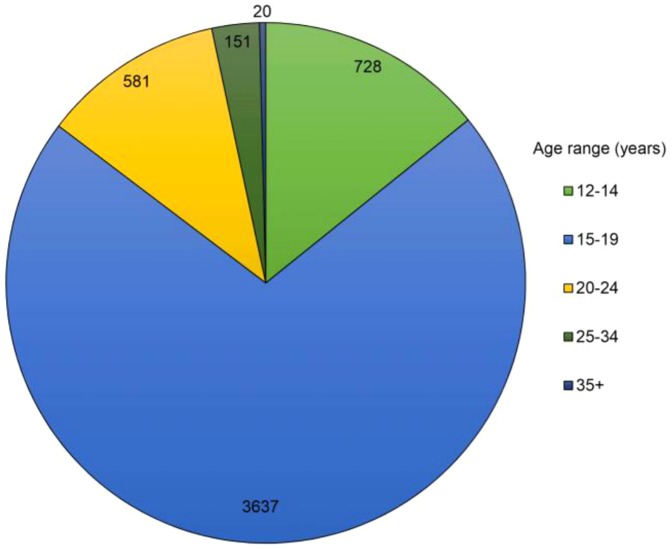
Proportions of VMMC performed by age group.

Of the males undergoing VMMC: 728 (14.2%) were aged 12–14; 3637 (71.1%) were aged 15–19; 581 (11.4%) were aged 20–24; 151 (3.0%) were aged 25–34; and 30 (0.4%) were aged 35 and above (Figure 3). Thus, there was high demand for VMMC and the majority of circumcisions were performed in the target age group of 15–19 years, with the median age for the procedure at 16 years.

About two thirds (63.6% (3285/5165)) of volunteers completed a post-operative review 48 hours following circumcision. The seven-day follow-up review was completed on 49.8% (2570/5165) of the students and the 21 day review was conducted for 27.4% (1414/5165). The majority of students attended either the 48 hour or 7 day follow-up review, although a small number attended both. However it is a concern that there were instances when students were known to have been at school on the day of the review but did not attend. The nurses and other program staff remained in close contact with the teachers to identify any possible adverse events. It was not possible to review VMMC clients who attended the clinic from outside the catchment schools unless they came to the clinic themselves.

No intraoperative adverse events were reported and the number of adverse events reported following the procedure was low. In the first year, adverse events were reported following 7.2% (168/2315) of procedures performed and included normal post-operative swelling. In year two this post-operative swelling was not required to be reported, according to Department of Health standard operating procedures for monitoring, reporting, and management of medical male circumcision adverse events in South Africa, thereby reducing adverse events to 1.3% (36/2850) of procedures performed. Of these, 17 (0.6%) were classified as mild, 18 (0.6%) as moderate and one (0.04%) as severe (Table 1).

**Table 1:** Adverse Effects March 2012-February 2013.

Grade	Frequency n/N (%)	Details (n)
Severe	1/2850 (0.04%)	Serious infection (treated and resolved) (1)
Moderate	18/2850 (0.6%)	Swelling (9)
		Local infection (8)
		Not recorded (1)
Mild	17/2850 (0.6%)	Local infection (15)
		Swelling (1)
		Bruising (1)

Post-operative adverse effects for the second year of the VMMC pilot are shown ranked by grade. No intra-operative adverse effects were recorded.

Only 11.3% (586/5165) of males attended a follow up visit occurring at a minimum of six-months post VMMC procedure at which personal experience of VMMC was reviewed and HCT offered; all of these 586 requested HCT, with 0.2% (1/586) of these testing positive for HIV and therefore assumed to have seroconverted at some time after the procedure.

## Discussion

After development of an adolescent-friendly VMMC program specifically targeted at young males in school we found that acceptability of VMMC in rural Umgungundlovu was high. Particularly following consultation and engagement of concerned community members who played an active role in disseminating the compelling scientific evidence of VMMC studies and obtaining traditional-, school-, and student representative council- support for the launch of the CAPRISA VMMC service. Community consultations were critical in creating an effective enabling environment for the recruitment of adolescents for VMMC services, the foundation of support that ensured that the program continued for over two years with high levels of uptake. It is difficult to objectively assess the success of the intervention; a Department of Health survey in Tanzania demonstrated an approximately 1.5-fold increase in VMMC rates in 15–24 year old males in a previously low-VMMC region following a government awareness program [24]. Although no baseline data is available for adolescent uptake of VMMC services in this setting prior to this pilot, a comparatively impressive 5.4-fold increase in VMMC uptake was observed following demand creation strategies, from an average of 58 procedures/month among instant and early adopters, to a peak of 308 procedures/month on initiation of phase three of the demand creation strategy in March 2012.

Monitoring, review, and adaption led to the success of the VMMC demand creation strategies in schools, with the launch of both the short in-school awareness sessions and the peer recruitment system dramatically increasing service uptake. As the various aspects of the demand creation strategy were designed to be complementary to one another, it is not possible to quantify the effect of each individual component on VMMC uptake. Additionally uptake may have been affected by other external factors not part of this program. However, these results confirm previous reports concerning the importance in behaviour change interventions of: (i) interpersonal communication from a variety of sources; (ii) using pre-existing community organizations to aid implementation; and (iii) developing locally responsive programs [25].

Ongoing review led to the development of highly contextualized models of service delivery and clinic procedures in this setting in order to make them “adolescent friendly” and thus increase uptake. In particular, integrating clinic operations with school timetables heightened program success, although this also resulted in marked reductions in VMMC demand during examination periods and school holidays and posed some challenges with respect to optimization of the service provision, predominantly with clinic staffing. Estimating reliable coverage also proved challenging. Using the number of male students per school and the number of VMMC participants from each school we estimate that the highest level of coverage obtained is 47%. This figure may be an overestimate as a measure of school-based coverage, as some circumcisions were performed on non-school attending males; conversely, coverage could be underestimated as some students may have been circumcised at non-CAPRISA clinics. Further, considerable variability in coverage was observed between schools. Future studies to understand the causes of such variation, as well as to determine maximal VMMC coverage levels, are ongoing and will be key in informing the feasibility of wide-scale implementation of a school-based VMMC program. Further work will also be required to improve linkage to care, and to reach more vulnerable adolescent populations who have dropped out of educational settings [26], [27].

The results of pre-VMMC HCT confirm previous reports from KwaZulu-Natal that HIV prevalence in males remains low until 25 years, and highlight the importance and potential cost-effectiveness of directing VMMC as part of combined prevention interventions at such males before entry into high risk periods and during a time when they seem most willing to undergo the procedure. It should be noted that these figures do not represent population age-specific HIV prevalence, given selection bias in uptake of HCT but are consistent with available data on HIV prevalence in males in this community.

High schools in low male circumcision, high HIV risk settings provide a convenient venue for reaching large numbers of young males who would benefit most from VMMC services which over time could become normative. As this is a once-off surgical procedure, service provision could be integrated into regular school visits to maintain high coverage rates and integrated into comprehensive school based sexual reproductive health services. This pilot adolescent-friendly demand creation and targeting strategy demonstrated acceptability, feasibility and success in this setting, imperatives to successful scale-up to reduce disease burdens.
